# Seasonal Ecology and Behavior of an Endangered Rainforest Frog (*Litoria rheocola*) Threatened by Disease

**DOI:** 10.1371/journal.pone.0127851

**Published:** 2015-05-19

**Authors:** Elizabeth A. Roznik, Ross A. Alford

**Affiliations:** School of Marine and Tropical Biology, James Cook University, Townsville, Queensland, Australia; Vanderbilt University School of Medicine, UNITED STATES

## Abstract

One of the most devastating wildlife diseases ever recorded is chytridiomycosis, a recently emerged amphibian disease that is caused by the chytrid fungus *Batrachochytrium dendrobatidis*. Understanding, predicting, and managing the impacts of chytridiomycosis on any amphibian species will require detailed information on its ecology and behavior because this pathogen is transmitted by contact with water or other individuals, and pathogen growth rates are thermally sensitive. The common mistfrog (*Litoria rheocola*) is an endangered tropical rainforest frog that has declined due to chytridiomycosis. We tracked *L*. *rheocola* during the winter (cool/dry) and summer (warm/wet) seasons at a low- and high-elevation site. We found that seasonal differences in environmental temperatures and frog behavior should render this species most vulnerable to *B*. *dendrobatidis* during cooler months and at higher elevations, which matches observed patterns of infection prevalence in this species. During winter, frogs moved shorter distances than during summer, and they spent less time in vegetation and more time in the stream, which should increase exposure to aquatic *B*. *dendrobatidis* zoospores. At a low-elevation site (40 m ASL), estimated body temperatures were within the optimal range for *B*. *dendrobatidis* growth (15-25°C) most of the time during winter, but they reached temperatures above this threshold frequently in summer. At a higher elevation (750 m ASL), estimated body temperatures were within the range most favorable for *B*. *dendrobatidis* year-round, and did not exceed 25°C, even during summer. Our study provides the first detailed information on the ecology and behavior of *L*. *rheocola* and suggests ecological mechanisms for infection dynamics that have been observed in this endangered species.

## Introduction

Amphibians have experienced rapid population declines and species extinctions in recent decades, and one-third of extant amphibians are classified as threatened [1]. Although there are numerous causes for these losses, including land use change, contaminants, overexploitation, introduced species, and climate change [2,3], emerging infectious diseases pose a great threat to global amphibian diversity [2–4]. One of the most significant wildlife diseases ever recorded is chytridiomycosis, a recently emerged disease that is caused by the chytrid fungus *Batrachochytrium dendrobatidis*; it has caused severe amphibian declines and extinctions in many regions of the world [2,5]. This parasitic fungus attacks the skin cells of amphibians and disrupts their osmoregulatory and transport functions, altering electrolyte concentrations in the blood, which can ultimately cause cardiac arrest if the fungal population on the host reaches a high density [6].

In many regions, amphibians are infected by *B*. *dendrobatidis* year-round, but they are often most vulnerable during cooler months and at higher elevations, particularly in tropical regions [7–9]. These patterns reflect the strong dependence of *B*. *dendrobatidis* on environmental conditions; this pathogen is highly sensitive to desiccation [10] and its growth and survival rates are strongly influenced by temperature (15–25°C is optimal and >28°C is lethal in culture [11,12]). Because of these environmental constraints, the prevalence and intensity of infections in amphibians, as well as mortality rates due to chytridiomycosis, often vary seasonally [7–9,13]. The behavior of amphibians can also influence their vulnerability to *B*. *dendrobatidis* by affecting rates of transmission and the buildup of infections on their skin. Even closely related frog species that occur at the same sites can have very different patterns of movement, habitat use, and social behavior, all of which can influence susceptibility to *B*. *dendrobatidis* [14,15]. Because aquatic fungal zoospores are transmitted by contact with infected individuals or with contaminated water [16], species that form aggregations or spend more time in water are more likely to be exposed to *B*. *dendrobatidis* [14,17]. Additionally, species and individuals that use microclimates that are cooler (<25°C), and thus more favorable to *B*. *dendrobatidis* growth and survival, are more likely to develop and maintain infections [18,19].

Although amphibians are one of the most threatened groups of vertebrates, their conservation is often hindered by a lack of basic ecological knowledge. This is particularly true for tropical stream-breeding species, which have experienced more numerous and severe declines than any other amphibian taxa [1,20,21]. Many of these species occur in remote, montane areas that are difficult to access, and consequently, little is known about their ecology and behavior. One such species is the common mistfrog (*Litoria rheocola*), an IUCN Endangered species [22] that occurs near rocky, fast-flowing rainforest streams in northeastern Queensland, Australia [23]. *Litoria rheocola* is a small treefrog (average male body size: 2.0 g, 31 mm; average female body size: 3.1 g, 36 mm [20]). By the mid-1990s, chytridiomycosis had extirpated this species at higher elevations (>400 m ASL) throughout its geographic range [20,24]; however, some populations have subsequently recovered or recolonized these areas [25] and are currently coexisting with the pathogen [26]. Habitat modification and fragmentation also threaten *L*. *rheocola* [23,27]. Approximately 20% of historical tropical rainforest in northeastern Queensland was cleared by 1983; this was most extensive at low elevations (<80 m ASL), where over 50% was cleared [28]. Although most remaining rainforest is now protected, small-scale clearing still occurs in non-protected areas [28,29]

Very little is known of the ecology and behavior of *L*. *rheocola*. Individuals of this species call and breed year-round, although reproductive behavior decreases during the coolest weather [19,23]. Their eggs are deposited in gelatinous masses beneath rocks in fast-flowing water, the tadpoles hatch and feed on algae growing on rocks in riffles, and adults feed on a wide range of invertebrates [23]. Knowledge of their behavior is based only on observations of individuals during nocturnal stream surveys [30] and in field enclosures [31]. We used harmonic direction finding [32,33] to track individual *L*. *rheocola* and study patterns of movement, microhabitat use, and body temperatures during winter and summer. The goal of our study was to understand the behavior of *L*. *rheocola*, and how it is affected by season and by sites that vary in elevation. The prevalence of *B*. *dendrobatidis* infection in *L*. *rheocola* is highest during winter and at higher-elevation sites throughout the year [9]. Therefore, we predicted that frogs would behave in ways that increase infection risk more during winter than summer, including moving less and spending more time near the stream and in cooler microhabitats. We also predicted that microhabitat temperatures would be inversely related to elevation in both seasons. We provide the first detailed information on the ecology and behavior of *L*. *rheocola* and suggest ecological mechanisms for observed patterns of infection dynamics. Our study is an example of how detailed ecological information can be collected on an amphibian, which can be used in combination with information from other sources to enhance conservation efforts.

## Materials and Methods

### Ethics statement

Protocols involving animals were approved by the Animal Ethics Committee at James Cook University (approval A1316). The Queensland Department of Environment and Resource Management also approved all animal protocols and provided permission to work at all field locations (permit WITK03070508).

### Study sites

We conducted our study at two rainforest streams that differed substantially in elevation; Frenchman Creek (40 m ASL; 17.307°S, 145.922°E) and Windin Creek (750 m ASL; 17.365°S, 145.717°E) are both located in Wooroonooran National Park, Queensland, Australia. Both streams are surrounded by tropical rainforest, characterized by dense vegetation, including large trees (10 m in height), vines, epiphytes, shrubs, and herbaceous plants. The streams vary in width (5–20 m), contain pools, runs, riffles, and waterfalls, and the streambeds consist of rocks that range in size from small pebbles to large boulders (10 m in diameter). At each site, we captured and tracked frogs along a 400-m section of stream over a three-week period during the winter (cool/dry season) in 2009 (Frenchman Creek: 13 July—6 August; Windin Creek: 18 August—9 September), and the summer (warm/wet season) in 2010 (Frenchman Creek: 20 January—9 February; Windin Creek: 11 February—3 March).

### Tracking

For the present study, we examined data only on tracked frogs that were not infected by *B*. *dendrobatidis* because infection by this pathogen can alter amphibian behavior [19,34–36], and we were interested in how the behavior of uninfected frogs could influence infection susceptibility. To prevent disease transmission between frogs during handling, each frog was captured in an unused plastic bag worn as a glove, and was handled only while wearing a new pair of disposable vinyl gloves. Each frog was sampled for infection by *B*. *dendrobatidis* at first capture, and a second sample was taken from recaptured frogs at the end of the study period to determine whether they had become infected. We swabbed the ventral surface and all four feet of each frog with a sterile rayon swab, covering these areas twice. Swab samples were analyzed using real-time quantitative PCR assays [37]. Frogs that tested positive at the start and/or end of our study were excluded from our dataset.

We tracked a total of 76 uninfected frogs: 42 during winter (Frenchman Creek: N = 30, Windin Creek: N = 12) and 34 during summer (Frenchman Creek: N = 20, Windin Creek: N = 14). Following capture, we recorded each frog’s sex (using presence/absence of distinct nuptial pads), body mass, and snout-urostyle length. We included both males and females in all analyses because females only represented a small proportion of the individuals that we tracked (9%, N = 7), and were not overrepresented in any tracking period (maximum of N = 3 per tracking period); therefore, any gender effects should not be confounded with treatment effects.

After each frog was processed following capture, it was immediately fitted with an external tracking device. Because *L*. *rheocola* are too small to carry radiotransmitters, we tracked frogs using harmonic direction finding [32,33]. Tracking devices had minimal effects on frog movement behavior [38]. We built tracking devices using SOT-323 surface-mount zero-bias Schottky detector diodes (Agilent Technologies, Forest Hill, VIC, Australia) attached to a belt made of silicone tubing [39]. The tubing was cut to length so it just encircled the frog’s inguinal region (waist), and a length of fine cotton thread was passed through the tubing and tied. The combined mass of the tracking device and belt never exceeded 8% of the frog’s body mass, which is below the recommended maximum 10% transmitter-to-body-mass ratio for amphibians [40]. We excluded all data collected during the 24-hr period following attachment of tracking devices due to potential short-term behavioral effects of handling, which are unlikely to persist after the first night of tag attachment [32,33].

We tracked frogs using RECCO detectors (models R4 and R8, RECCO Avalanche Rescue System, Lidingö, Sweden). These hand-held devices act as both transmitters and receivers; they emit a continuous signal that is absorbed and re-emitted at a higher frequency by the diodes. We attempted to locate frogs once during each day (10:00–17:00) and once each night (20:00–03:00) throughout the tracking period. To do this, we walked slowly along the center and edges of the stream and used the detector to scan all areas potentially used by frogs, including rocks in the stream and vegetation along the stream edge. At the end of the tracking period, we removed the tracking devices from all recaptured frogs (N = 19); the harnesses were designed to fall off the remaining frogs after approximately 21 days as the fine cotton thread deteriorated. Using harmonic direction finding to locate animals is not as effective as radiotelemetry; the tracking detector typically has a maximum detection range of 15 m at rainforest streams and cannot penetrate rock [33]. However, this was unlikely to cause a bias toward shorter movements in our study; *L*. *rheocola* has strong site fidelity and when a frog was not found on a particular survey (or surveys), it was almost always subsequently found less than 2 m from its most recent known location. This suggests that frogs were moving short distances and sheltering beneath rocks when we could not detect them. Because we were not able to locate all frogs on all surveys, sample sizes vary among analyses, based on available data. We attempted to meet the assumptions of all statistical analyses, but we used raw data in cases when transformations did not result in normal distributions because ANOVA is robust to deviations from normality [41].

### Movements

We examined frog movements at two scales: daily displacement of frogs, and total displacement of frogs over the three-week study period. Each time we located a frog, we marked its location using flagging tape, and recorded its distance along the transect. We also measured the frog's height above the stream, and its horizontal distance from the stream edge. To investigate the daily movements of frogs, we measured the distances moved by frogs between consecutive locations; this includes movement from each nocturnal perch site to the subsequent diurnal shelter site, and from each diurnal shelter site to the subsequent nocturnal perch site. *Litoria rheocola* is a treefrog, and individuals move along and at right angles to the stream and also climb up and down vegetation; therefore, they use all three dimensions of space, with their directions of movement largely unconstrained in the horizontal plane but largely restricted to movements up and down individual plants in the vertical direction. Because of these movement patterns, we recorded horizontal and vertical displacement from the previous location separately (to the nearest 5 cm). Movement distances were calculated only when individuals were located on consecutive surveys (i.e., day to night, or night to day); when frogs were not located on consecutive surveys, movement distances for the time interval concerned were recorded as missing values and were not included in any analyses. We also determined the probability of movement from day or night locations by calculating the movement probability for each frog (number of times each individual frog moved between consecutive locations divided by the total number of times the frog was located). We used a two-way MANOVA to analyze the distances moved by individual frogs between day and night locations, and the probability of moving from these locations. We used season (winter or summer) and site (Frenchman Creek or Windin Creek) as independent variables, and the median horizontal distance between locations, median vertical distance between locations, and movement probability of each frog as dependent variables.

We examined longer-term movements of frogs by determining their displacement along the stream during our three-week study period. For each frog, we calculated the difference between the minimum and maximum distances along our stream transect at which the frog was observed. We analyzed these data using a two-way ANOVA with season and site as independent variables, and total displacement of each frog as the dependent variable.

We also studied the positions of frogs in relation to the stream to determine whether their proximity to the stream varied by season and site. We used two-way MANOVAs to analyze these data. Season and site were independent variables, and median height above stream, and median horizontal distance from the stream edge of each frog were dependent variables. Separate analyses were performed for day and night locations.

### Body temperatures

We used physical models that mimic the thermal and hydric properties of frogs [42,43] to estimate frog body temperatures and examine their distributions over time. Models were placed in each unique location used by each frog. Frogs typically spent the entire day or night in the same location; therefore, placing models in the locations at which we observed frogs allowed us to accurately estimate the temperatures experienced by frogs over time [43]. We placed models in diurnal locations used by frogs to measure temperatures at 30-min intervals from 07:00 to 18:30, and we placed models in nocturnal locations used by frogs to measure temperatures from 19:00 to 06:30. Each frog-shaped model was made of three percent agar and contained an embedded Thermochron iButton temperature datalogger (Maxim Integrated Products, California, USA; factory-calibrated and accurate to ± 0.5°C) that was waterproofed during summer tracking to prevent failure from moisture damage [44]. These models lose and gain water at rates similar to frogs, and temperatures obtained from these permeable models are closely correlated with *L*. *rheocola* body temperatures [43].

We used all thermal data collected for each frog to calculate the proportion of its body temperatures that occurred in temperature categories that are relevant to the growth of *B*. *dendrobatidis* in culture: <15°C, 15–25°C, and >25°C [11,12]. Growth of *B*. *dendrobatidis* in northeastern Queensland is fastest between 15°C and 25°C in culture, and is slower outside of this temperature range [12]. Although temperatures above 28°C are lethal to *B*. *dendrobatidis* in culture and on amphibian hosts [12], we did not establish a separate category for these readings because they were rare during our study; most frogs had no temperature readings above 28°C. We used a two-way MANOVA to examine frog body temperatures; season (winter or summer) and site (Frenchman Creek or Windin Creek) were used as independent variables, and the proportion of temperatures in each temperature category were the dependent variables.

### Microhabitat use

Each time we located a frog, we recorded the substrate that the ventral surface of the frog was contacting. We defined four substrate categories: vegetation, rock, leaf litter, and soil/coarse woody debris. We also recorded whether the substrate was dry or wet (from the stream or rain), and whether the frog was in a sheltered or exposed position. For each frog, we calculated the proportion of locations in each substrate category, the proportion of locations that were wet, and the proportion that were sheltered. We performed separate calculations for day and night locations. To analyze microhabitat data, we used multi-response permutation procedures (MRPP) and Monte Carlo re-sampling with 10,000 iterations (using Blossom statistical software [45]). We performed the analysis in a stepwise manner, testing for differences between seasons (winter and summer), time of day (day and night), and sites (Frenchman Creek and Windin Creek), in that order. If a difference was detected between groups, the next analysis was performed on each of those groups separately. For all analyses, the dependent variables were the proportion of locations on a wet substrate, proportion of locations in a sheltered position, and the proportion of locations in each of the four substrate categories described above.

We also estimated the rate of evaporative water loss in each microhabitat used by each frog by weighing the permeable physical model (described above) to the nearest 0.1 g immediately before and after placement in each frog location, and calculating the proportion of mass lost over 48 hr [42,46]. For each frog, we calculated the median proportion of model mass lost, using all of the models placed in locations used by that frog. Separate calculations were performed for day and night models. We analyzed our data using a two-way ANOVA with season (winter or summer) and site (Frenchman Creek or Windin Creek) as independent variables, and the median proportion of model mass lost over 48 hr as the dependent variable. Separate analyses were performed for models corresponding to day and night locations.

### Perch-site selection

We studied nocturnal perch-site selection by frogs during winter and summer. Because of obvious seasonal differences in selected perch sites, we used different approaches for each season. During winter, we measured the following characteristics of vegetation used by frogs at each perch site: distance from the stream edge, distance to the nearest riffle, plant height, area covered by the plant’s canopy (average length × width), stream depth, and number of rocks within 1 m. We also measured these same characteristics for vegetation that was available to frogs, but was not used by frogs during our study; we selected the nearest plant to the stream every 10 m along both sides of a 200-m section of stream for measurement. To determine whether frogs selected perch sites that were different from available perch sites, we analyzed our data using a two-way MANOVA. We used perch type (used or available) and site (Frenchman Creek or Windin Creek) as independent variables, and the six characteristics described above as dependent variables.

During summer, frogs were often observed in taller vegetation than during winter, especially in taller trees. To determine whether this seasonal difference was statistically significant, we compared the height of vegetation that frogs perched on during the two seasons. We used a two-way ANOVA with season and site as independent variables, and vegetation height as the dependent variable. To understand whether frogs selected trees that were significantly different from available trees during summer, we measured the following characteristics of all trees used by frogs: tree height, height of the lowest branch, and diameter at breast height (DBH). We measured these same characteristics for trees that were available to frogs, but not used by frogs during our study; we selected all unused trees within 3 m of the stream along one side of a 200-m section of stream for measurement. To determine whether frogs selected trees that were different from available trees, we analyzed our data using a two-way MANOVA. We used tree type (used or available) and site as independent variables, and tree height, height of the lowest branch, and DBH as dependent variables.

## Results

### Movements

**Fig 1:**
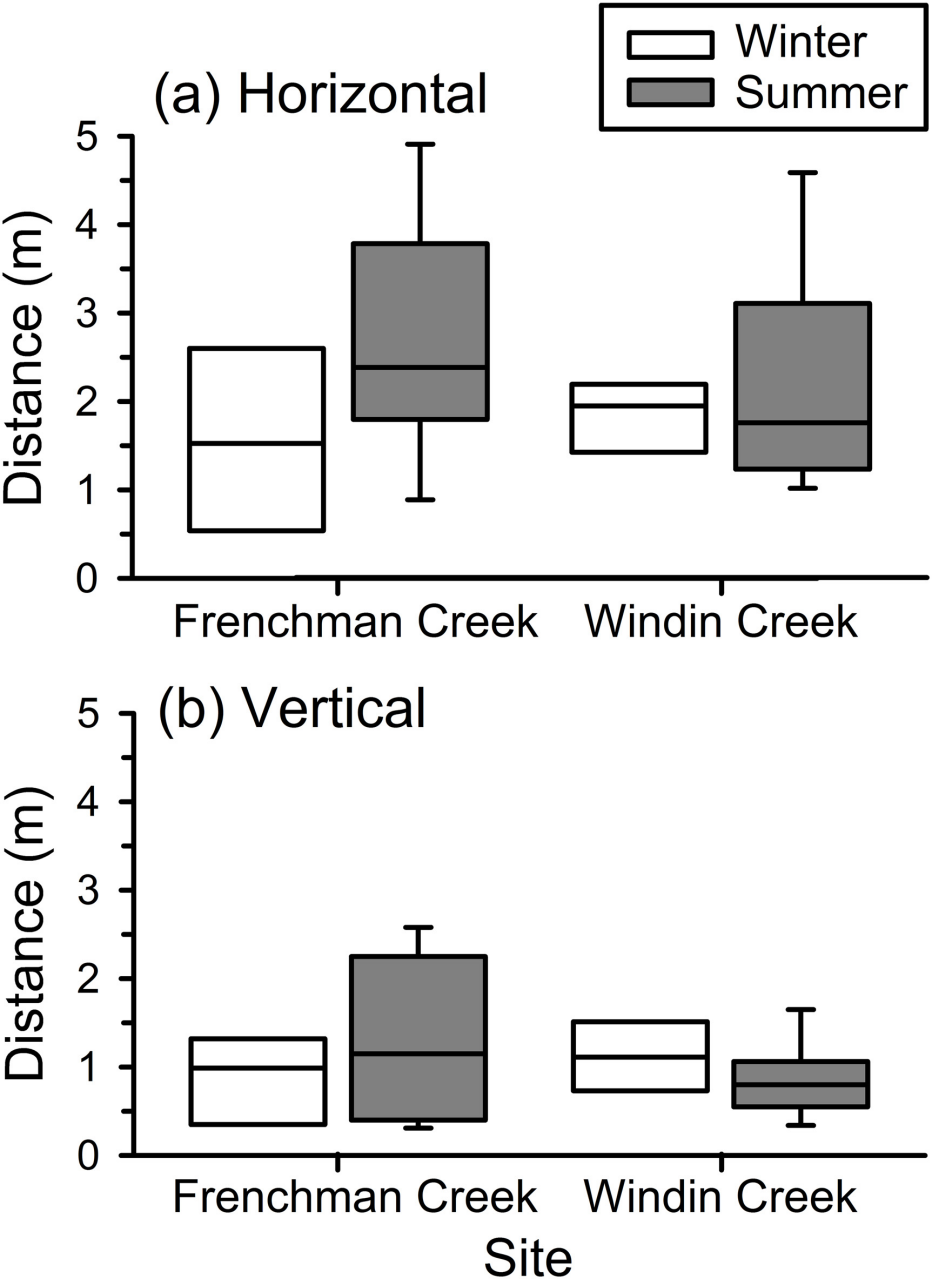
Distances moved by common mistfrogs (*Litoria rheocola*). Shown are box plots of (a) horizontal and (b) vertical distances moved frogs between day and night locations at two rainforest streams (Frenchman Creek and Windin Creek) during the winter (cool/dry season) and summer (warm/wet season).

Daily movement patterns were significantly affected by season (MANOVA, F_3,27_ = 3.192, P = 0.039), but not by site (MANOVA: F_3,27_ = 0.351, P = 0.789), and there was a marginally significant interaction between season and site (MANOVA: F_3,27_ = 3.036, P = 0.046). One-way ANOVAs on each variable indicated that the only significant univariate result was that frogs showed a seasonal difference in horizontal movements between day and night locations (F_1,29_ = 6.980, P = 0.013), although the seasonal difference in vertical movements between day and night locations was nearly significant (F_1,29_ = 3.619, P = 0.067). The overall pattern was that frogs moved significantly longer horizontal distances between diurnal shelter sites and nocturnal perch sites during summer than in winter at both sites (Fig 1). During summer, frogs moved longer vertical distances at Frenchman Creek, and made slightly shorter vertical movements at Windin Creek (Fig 1). The probability of movement did not differ significantly in any of these analyses (all P ≥ 0.129); on average, frogs moved away from both day and night locations 84% of the time.

The length of stream used by frogs during our three-week study period was influenced significantly by the interaction of season and site (F_1,60_ = 4.919, P = 0.320). At Windin Creek, the length of stream used by frogs during summer was 78.9% longer than that used during winter, but at Frenchman Creek, the length of stream used did not differ significantly between seasons. The median stream length used by frogs at Windin Creek was 2.0 m (range: 0–16 m) during winter, and 9.5 m (range: 0–57 m) during summer, and the median (and range) across seasons was 4.0 m (0–50 m) at Frenchman Creek.

**Fig 2:**
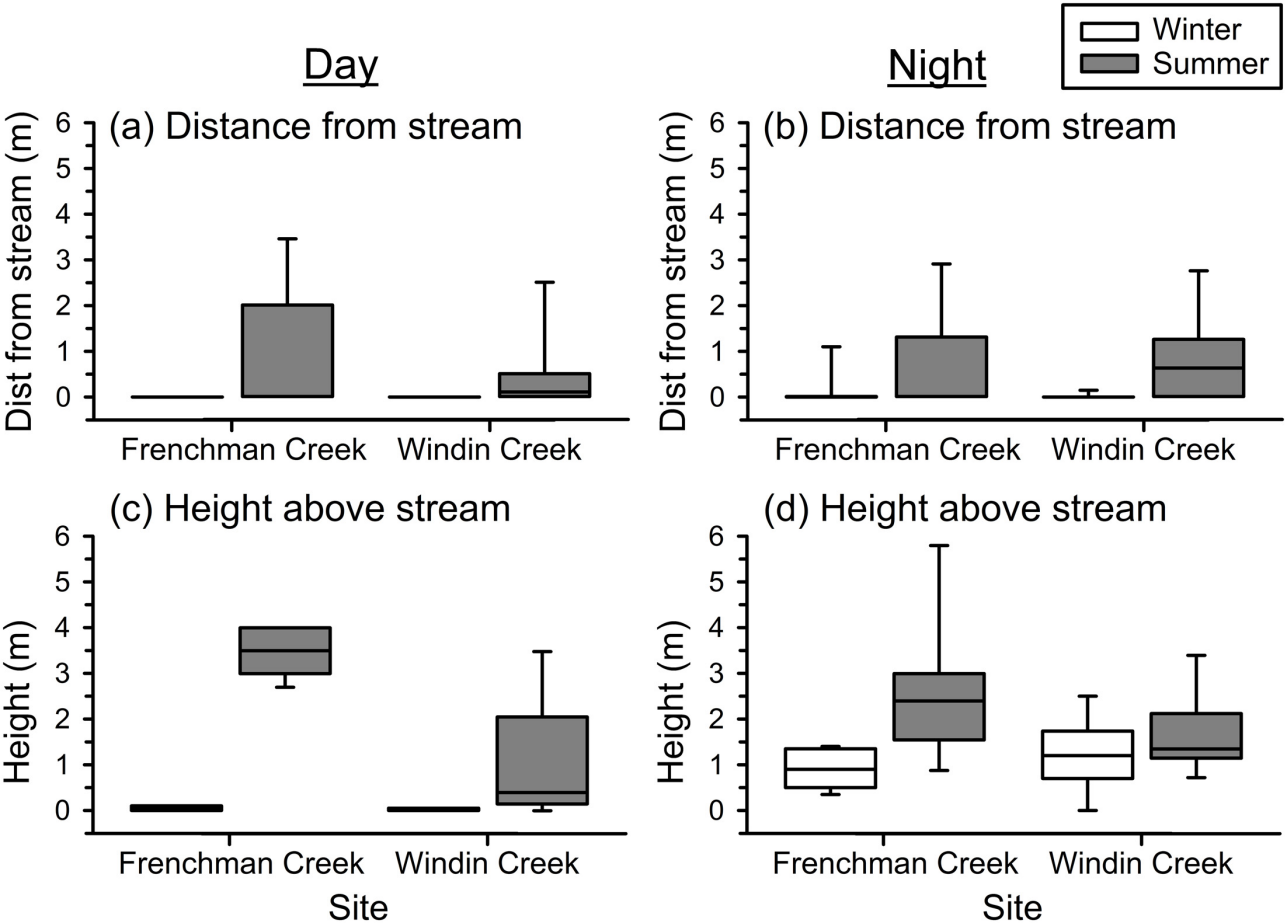
Proximity of common mistfrogs (*Litoria rheocola*) to the stream. Shown are box plots of (a-b) horizontal distances from the stream edge, and (c-d) vertical heights above the stream where frogs were located during the day and night at two rainforest streams (Frenchman Creek and Windin Creek) during the winter (cool/dry season) and summer (warm/wet season).

The proximity of frogs to the stream during the day differed significantly between seasons (MANOVA: F_2,35_ = 35.504, P < 0.001) and sites (MANOVA: F_2,35_ = 9.783, P < 0.001), and there was a significant interaction between season and site (MANOVA: F_2,35_ = 10.068, P < 0.001). Frog position relative to the stream during the night also differed significantly between seasons (MANOVA: F_2,40_ = 5.571, P = 0.007), but not between sites (MANOVA: F_2,40_ = 1.134, P = 0.332); however, there was a significant interaction between season and site (MANOVA: F_2,40_ = 5.312, P = 0.009). Results from one-way ANOVAs indicated that frog perch sites were higher above the stream during summer than winter, and that the extent of this difference depended upon site; heights were similar at both sites during winter, but tended to be higher at Frenchman Creek than Windin Creek during summer during both the day (season × site: F_1,36_ = 18.322, P < 0.001; Fig 2) and night (season × site: F_1,41_ = 3.855, P = 0.056; Fig 2). Perch sites were higher at night than during the day during winter, but were similar in height during the day and night during summer (Fig 2). The horizontal distance from stream only differed significantly between seasons during the night (F_1,41_ = 6.957, P = 0.012), although there was a trend during the day (F_1,36_ = 3.489, P = 0.070); frogs were observed farther from the stream during summer than winter, and this pattern did not differ between sites during the day (season × site: F_1,36_ = 0.211, P = 0.649) or night (season × site: F_1,41_ = 0.154, P = 0.697; Fig 2). On average, frogs were observed 0.10 m (range: 0–1.80 m) from the stream during winter, and 0.74 m (range: 0–3.75 m) from the stream during summer.

### Body temperatures

**Fig 3:**
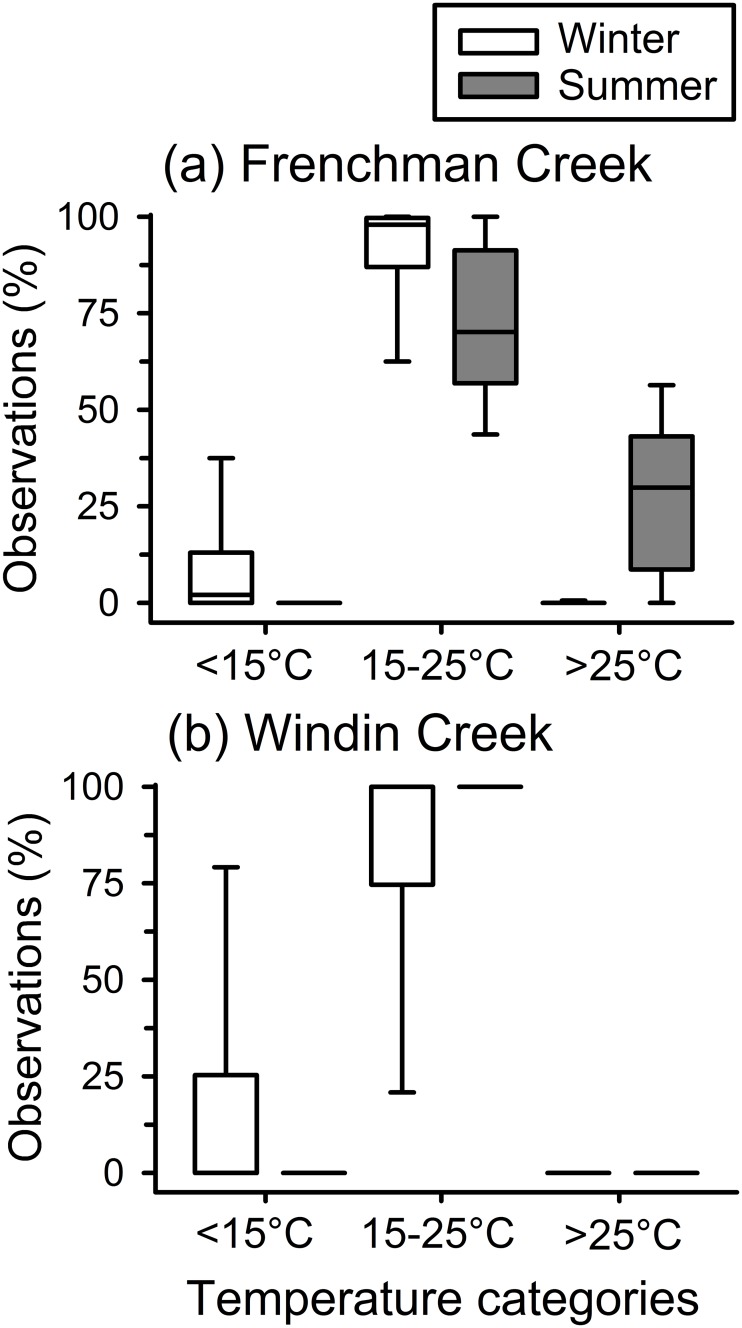
Distribution of estimated body temperatures of common mistfrogs (*Litoria rheocola*) within categories relevant to *Batrachochytrium dendrobatidis* growth in culture (<15°C, 15–25°C, >25°C). Temperatures were estimated using agar models at two rainforest streams (Frenchman Creek and Windin Creek) during the winter (cool/dry season) and summer (warm/wet season).

**Fig 4:**
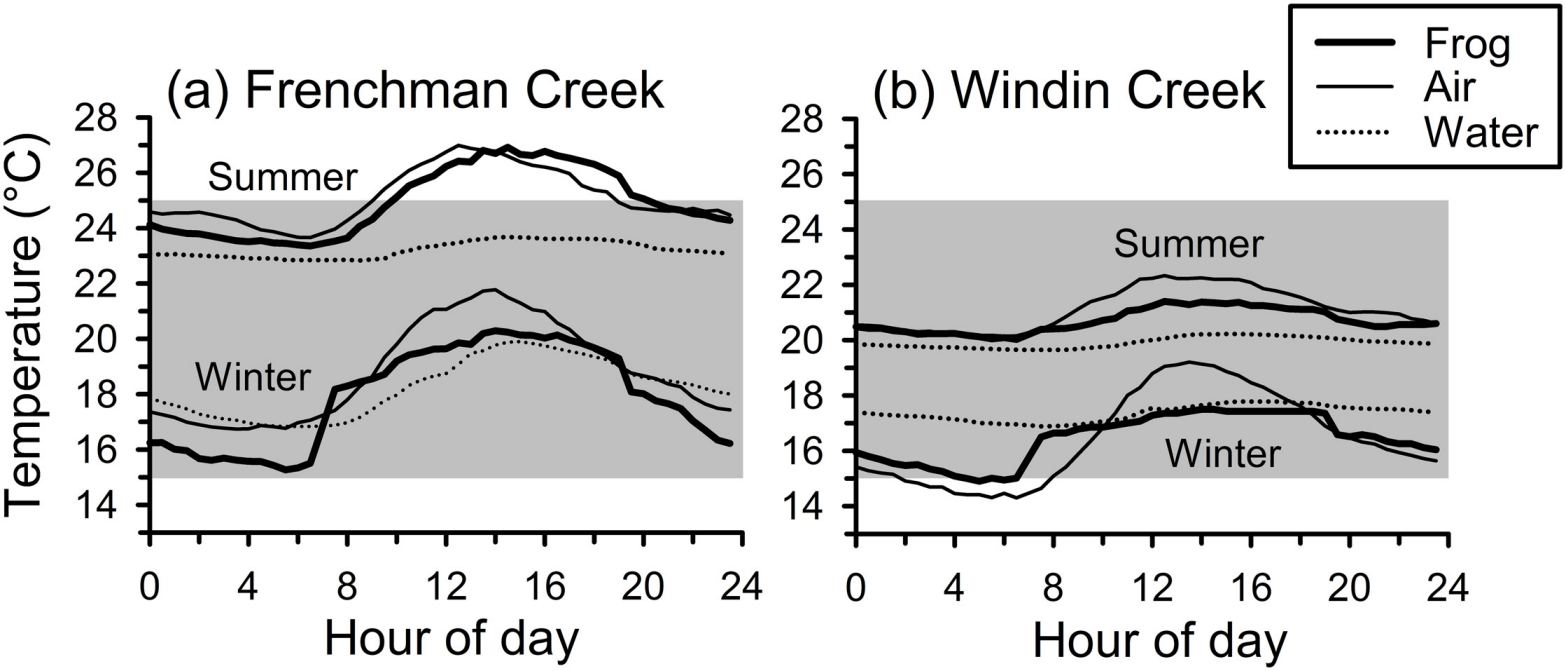
Mean estimated body temperatures of common mistfrogs (*Litoria rheocola*) over the 24-hr diel period. Temperatures were estimated using agar models at two rainforest streams (Frenchman Creek and Windin Creek) during the winter (cool/dry season) and summer (warm/wet season). Also shown are the mean ambient air and water temperatures at each site during each season. The optimal thermal range for *Batrachochytrium dendrobatidis* growth in culture (15–25°C) is shaded.

Frog body temperatures estimated using our models were warmer during summer than in winter, and were warmer at Frenchman Creek than at Windin Creek during both seasons (Figs 3 and 4). The distribution of estimated frog body temperatures within categories relevant to *Batrachochytrium dendrobatidis* growth in culture (<15°C, 15–25°C, >25°C) differed significantly between seasons (MANOVA: F_3,44_ = 7.852, P < 0.001), and sites (MANOVA: F_3,44_ = 5.203, P = 0.004), and there was a significant interaction between season and site (MANOVA: F_3,44_ = 5.161, P = 0.004; Figs 3 and 4). In follow-up ANOVAs, the season × site interaction was significant for the proportion of temperatures that were 15–25°C (F_1,46_ = 11.938, P = 0.001), and those that were above 25°C (F_1,46_ = 15.317, P < 0.001), but not those that were below 15°C (F_1,46_ = 0.847, P = 0.362). The proportion of temperatures below 15°C was higher during winter than during summer at both sites (Fig 3). At Frenchman Creek, frogs spent more time in temperatures optimal for *B*. *dendrobatidis* growth (15–25°C) during winter than summer, and they reached temperatures above this threshold (>25°C) rarely during winter, but frequently during summer (Figs 3 and 4). At Windin Creek, frogs were within temperatures optimal for *B*. *dendrobatidis* more often during summer than in winter, and they did not reach temperatures above this threshold during either season (Figs 3 and 4).

### Microhabitat use

Microhabitats used by frogs were affected significantly by season (δ = 3.102, P < 0.001) and by time of day during both winter (δ = 2.505, P < 0.001) and summer (δ = 2.744, P < 0.001; Table 1). Microhabitat use was not significantly different between the two sites except during the day in summer (δ = 2.514, P < 0.001; all other P > 0.079). During winter, frogs typically sheltered between moist rocks in the stream bed during the day, and perched on dry, exposed vegetation at night (Table 1). During summer, all substrates used by frogs were wet due to frequent rainfall; during the day, frogs at Frenchman Creek typically used vegetation, and frogs at Windin Creek used vegetation, leaf litter, and rocks, and during the night, frogs at both sites typically perched on vegetation (Table 1).

**Table 1:** Characteristics of microhabitats used by common mistfrogs (*Litoria rheocola*) during the day and night at two rainforest streams (Frenchman Creek and Windin Creek) during the winter (cool/dry season) and summer (warm/wet season).

Characteristic	Winter	Winter	Summer	Summer	Summer
	Both sites	Both sites	Frenchman Creek	Windin Creek	Both sites
	Day	Night	Day	Day	Night
**Wet**	86.4 (0–100)	9.8 (0–80.0)	100 (100–100)	100 (100–100)	100 (100–100)
**Sheltered**	86.5 (0–100)	11.0 (0–100)	10.0 (0–50.0)	42.1 (0–100)	1.4 (0–25.0)
**Vegetation**	7.9 (0–66.7)	87.0 (0–100)	89.2 (50.0–100)	35.4 (0–100)	96.0 (75.0–100)
**Rock**	67.9 (0–100)	11.8 (0–100)	0 (0–0)	25.2 (0–100)	1.7 (0–25.0)
**Leaf litter**	22.8 (0–100)	1.3 (0–25.0)	9.3 (0–50.0)	34.7 (0–100)	0.6 (0–12.5)
**Soil/wood**	1.4 (0–33.3)	0 (0–0)	1.5 (0–14.3)	4.6 (0–33.3)	1.7 (0–25.0)

Shown are the mean percentages (and ranges) of microhabitats that were wet (from rain or stream water), sheltered, and characterized by vegetation, rock, leaf litter, and soil/coarse woody debris (wood). Means were calculated using the median value for each individual frog. Only categories that are significantly different from each other (P < 0.05) are shown.

Evaporative water loss (estimated using physiological models) in diurnal microhabitats used by frogs differed significantly between seasons (F_1,39_ = 24.915, P < 0.001) and sites (F_1,39_ = 26.565, P < 0.001), and there was a significant interaction between the effects of season and site (F_1,39_ = 18.749, P < 0.001). Evaporative water loss in nocturnal microhabitats differed significantly between seasons (F_1,54_ = 7.910, P = 0.007) and sites (F_1,54_ = 10.930, P = 0.002), but the interactions between these effects was not significant (F_1,54_ = 2.558, P = 0.116). Microhabitats used by frogs during the night were significantly drier during winter than summer, but diurnal microhabitats were significantly drier during summer than winter. Overall, microhabitats used by frogs during the day and night at Frenchman Creek were significantly drier than those used at Windin Creek.

### Perch-site selection

During winter, frogs selected nocturnal perch sites that differed significantly from our sample of available perch sites (MANOVA: F_6,171_ = 18.082, P < 0.001; Table 2). Perch site use also differed significantly between sites (MANOVA: F_6,171_ = 6.544, P < 0.001), although this difference appears to be driven only by differences in water depth below perch sites (one-way ANOVA: F_1,176_ = 36.226, P < 0.001; all other P ≥ 0.450). Additionally, there was a significant interaction between perch type (used or available) and site (MANOVA: F_1,172_ = 2.313, P = 0.036). The overall pattern was that, when compared to available vegetation, frogs selected vegetation that was significantly taller, had a significantly fuller canopy (i.e., was bushier), and was located significantly closer to riffles and closer to the stream edge (Table 2). Selected vegetation was also located along significantly shallower areas of the stream with more rocks (Table 2). These patterns were similar between sites; however, the difference between the average height of used and available vegetation was greater at Frenchman Creek than at Windin Creek (perch type × site: F_1,176_ = 7.918, P = 0.005), and the difference between the average water depth below perch sites was marginally greater at Windin Creek than at Frenchman Creek (perch type × site: F_1,176_ = 3.656, P = 0.057; Table 2).

**Table 2:** Results of separate one-way ANOVAs comparing characteristics of nocturnal perch sites that were available to and used by common mistfrogs (*Litoria rheocola*) at two rainforest streams (Frenchman Creek and Windin Creek) during winter (cool/dry season).

Characteristic	F_1,176_	P	Frenchman Creek	Frenchman Creek	Windin Creek	Windin Creek
			Used	Available	Used	Available
**Distance from riffle (m)**	36.076	<0.001	0 (0–0)	4.5 (0–30.0)	0.4 (0–10.0)	4.3 (0–20.0)
**Distance from stream (m)**	7.618	0.006	0.8 (0–4.3)	1.3 (0–3.6)	0.9 (0–3.1)	1.0 (0–2.7)
**Plant height (m)**	16.361	<0.001	2.6 (0.6–6.0)	1.6 (0.5–3.5)	2.1 (0.5–4.0)	1.9 (0.3–3.3)
**Canopy area (m** ^**2**^ **)**	6.749	0.010	4.0 (0.1–40.0)	1.7 (0.1–11.2)	3.4 (0.1–12.0)	2.4 (0.3–14.3)
**Water depth (cm)**	9.242	0.003	17.8 (10–30)	19.2 (5–50)	8.4 (5–35)	14.3 (5–35)
**Number of rocks**	54.579	<0.001	21.0 (0–55)	7.7 (0–32)	19.8 (0–55)	8.5 (0–25)

The mean value (and range) is shown for each perch-site characteristic at each site.

When we compared the height of vegetation used by frogs during winter and summer, we found a significant interaction between vegetation height and site (F_1,269_ = 11.299, P = 0.001). At Frenchman Creek, frogs used vegetation that was 1.3 m taller, on average, during summer than during winter, but at Windin Creek, average vegetation height was similar between the seasons (0.2 m difference). During summer, frogs often used trees as perch sites; the trees used by frogs were significantly different from available trees (MANOVA: F_3,318_ = 6.234, P = 0.0004). Overall, the characteristics of trees present at the two sites differed significantly (MANOVA: F_3,318_ = 12.006, P < 0.001); trees were taller (F_1.320_ = 19.334, P < 0.001) and larger in DBH (F_1.320_ = 4.006, P < 0.046) at Frenchman Creek than at Windin Creek. There was also a significant interaction between tree type (used or available) and site (MANOVA: F_3,318_ = 6.888, P = 0.0002). Frogs at Frenchman Creek selected trees that were significantly shorter than available trees (means: 5.8 m, and 9.2 m, respectively), whereas frogs at Windin Creek did not select trees that were significantly different in height than available trees (mean: 3.2 m; tree type × site: F_1.320_ = 5.466, P = 0.020). At both sites, frogs selected trees with significantly lower branches than those of available trees (F_1.320_ = 18.633, P < 0.001); the average height of the lowest branch of used and available trees was 2.3 and 5.0 m, respectively, at Frenchman Creek, and 2.1 and 6.9 m, respectively, at Windin Creek. Frogs did not select trees that differed significantly in DBH (F_1.320_ = 3.084, P = 0.080) from available trees; on average, the DBH of used trees was 9.5 cm at Frenchman Creek, and 3.2 cm at Windin Creek.

## Discussion

Our study provides the first detailed information on the ecology and behavior of the common mistfrog (*Litoria rheocola*), an IUCN Endangered species [22]. Overall, we found that *L*. *rheocola* are relatively sedentary frogs that are restricted to the stream environment, and prefer sections of the stream with riffles, numerous rocks, and overhanging vegetation (Table 2). Our study sites spanned a relatively wide elevational range, and despite large differences in environmental temperatures between sites (Fig 4), frog behavior was remarkably similar between low- and high-elevation streams. We found that seasonal differences in environmental temperatures and frog behavior should render this species most vulnerable to infection by *Batrachochytrium dendrobatidis* during cooler months and at higher elevations, which matches our predictions based on observed patterns of infection prevalence in this species [9].

Our data confirm that *L*. *rheocola* are active year-round, but their behavior varies substantially between seasons. During the summer, frogs moved longer distances between diurnal shelter sites and nocturnal perch sites (Fig 1) and spent more time away from the stream (Fig 2) than during winter. Frogs typically perched on vegetation during the day and night during summer, but in winter, frogs usually sheltered between wet rocks in the stream during the day, and climbed into vegetation above the stream at night (Table 1). Retallick [31] also found that juvenile and adult *L*. *rheocola* in field enclosures altered their behavior by season in similar ways; frogs used elevated perches more often in summer, and aquatic microhabitats more often during winter. Additionally, Hodgkison and Hero [30] observed more *L*. *rheocola* at the stream during warmer months, suggesting that during that period frogs used perch sites that were more exposed and elevated than those used during cooler months, when frogs were seen less frequently.

The seasonal differences in frog behavior that we observed should cause levels of exposure to *B*. *dendrobatidis* to be higher in winter than in summer because frogs spent more time in the stream in winter. Infectious *B*. *dendrobatidis* zoospores are aquatic, can survive in environmental reservoirs [47,48], and can be transmitted to frogs by contact with water [16]. Although *B*. *dendrobatidis* also may have alternative modes of transmission, aquatic transmission is probably the predominant mode in these stream-breeding frogs that permanently inhabit riparian habitats and breed throughout the year. We therefore suggest that the high frequency of contact between frogs and stream water during winter should lead to relatively high rates of pathogen transmission if the abundance of zoospores present in the stream is similar throughout the year or higher in winter. In contrast, frogs spent much more time in vegetation above the stream during summer, and thus should be less exposed to the pathogen during the summer season. The sedentary behavior of *L*. *rheocola* also may increase the vulnerability of this species to chytridiomycosis, particularly during winter, when movements are reduced. Zoospores released from an infected frog that remains within a very restricted area could build up in on its skin, or in its microhabitat, and re-infect the frog, thereby maintaining or increasing its fungal load [49], in a manner analogous to the accumulation of parasites that occurs in other sedentary taxa [50–52].

Once a frog is exposed to a *B*. *dendrobatidis* zoospore, its thermoregulatory behavior and the environmental conditions it experiences should play a major role in determining whether it develops an infection [18,19]. Rates of *B*. *dendrobatidis* survival and growth are strongly influenced by temperature and moisture; the fungus is highly sensitive to desiccation [10], and temperatures within 15–25°C are optimal in culture [11,12]. Our results suggest that seasonal differences in environmental temperatures and *L*. *rheocola* body temperatures should cause this species to be more likely to develop *B*. *dendrobatidis* infections during cooler months and at higher elevations (Figs 3 and 4); this matches observed patterns of infection prevalence [9]. At our low-elevation stream, the body temperatures of frogs should cause them to be more likely to develop infections during winter than summer. Although some low-elevation frogs occasionally attained body temperatures above 25°C during winter, most frogs regularly reached temperatures above this threshold during summer. High-elevation frogs should be more vulnerable to acquiring *B*. *dendrobatidis* infections than frogs at the low elevation because their temperatures were largely within the optimal range for the pathogen (15–25°C) year-round. This temperature range is based on the performance of *B*. *dendrobatidis* cultures maintained at constant temperatures [12], and fungal growth and survival could therefore differ under fluctuating conditions [53] and on frog hosts, particularly if infected frogs thermoregulate differently than uninfected frogs [18,19,54].

Detailed ecological studies are necessary to understand and conserve endangered species. Even closely related frog species that occur at the same sites can have very different patterns of movement and microhabitat use, and can also differ considerably in their vulnerability to disease-related declines [14,15,19]. For species threatened by *B*. *dendrobatidis*, it is important to study the behavior of uninfected individuals because this pathogen may alter the behavior of amphibians [19,34–36]. Our study provides detailed information on the movements, microhabitat use, and body temperatures of uninfected *L*. *rheocola*, and reveals how these behaviors differ by season and between sites varying in elevation. Seasonal differences in environmental conditions and frog behavior cause this species to be most vulnerable to *B*. *dendrobatidis* during cooler months and at higher elevations, providing an ecological mechanism for observed patterns of infection dynamics [9]. As with many stream-breeding frog species, females and juveniles are rarely observed because they spend more time away from the stream [15]; therefore, further study is necessary to understand behavioral differences between sexes and life stages, and the implications for disease risk.

## Supporting Information

S1 File Supporting data. (XLS)
